# Can Serum Cystatin C predict long-term survival in cardiac surgery patients?

**DOI:** 10.18632/aging.101403

**Published:** 2018-03-27

**Authors:** Valentina Rovella, Giulia Marrone, Mariarita Dessì, Michele Ferrannini, Nicola Toschi, Antonio Pellegrino, Maurizio Casasco, Nicola Di Daniele, Annalisa Noce

**Affiliations:** 1 Department of Medicine, Hypertension and Nephrology Unit, University Hospital Tor Vergata, Rome 00133, Italy; 2 PhD School of Applied Medical-Surgical Sciences, University of Rome Tor Vergata, Rome 00133, Italy; 3 Department of Laboratory Medicine, University Hospital Tor Vergata, Rome 00133, Italy; 4 Department of Biomedicine and Prevention, Medical Physics Section, University of Rome Tor Vergata, Rome 00133, Italy; ^5^Department of Radiology, Athinoula A. Martinos Center for Biomedical Imaging and Harvard Medical School, Boston, MA 02129, USA; ^6^Department of Cardiac Surgery, University Hospital Tor Vergata, Rome 00133, Italy; 7 Federazione Medico Sportiva Italiana, Rome 00196, Italy

**Keywords:** serum cystatin C, cardio-vascular mortality, serum creatinine, risk stratification, cardiac surgery, cardiovascular biomarker

## Abstract

Renal dysfunction is a risk factor for morbidity and mortality in cardiac surgery patients. Serum Cystatin C (sCysC) is a well-recognized marker of early renal dysfunction but few reports evaluate its prognostic cardio-vascular role. The aim of the study is to consider the prognostic value of sCysC for cardiovascular mortality. Four hundred twenty-four cardiac-surgery patients (264 men and 160 women) were enrolled. At admission, all patients were tested for renal function and inflammatory status. Patients were subdivided in subgroups according to the values of the following variables: sCysC, serum Creatinine (sCrea), age, high sensitivity-C Reactive Protein, fibrinogen, surgical procedures and Kaplan-Meier cumulative survival curves were plotted. The primary end-point was cardiovascular mortality. In order to evaluate the simultaneous independent impact of all measured variables on survival we fitted a multivariate Cox-Proportional Hazard Model (CPHM). In Kaplan-Meier analysis 124 patients (29.4%) reached the end-point. In multivariate CPHM, the only significant predictors of mortality were sCysC (p<0.00001, risk ratio: 1.529, CI: 1.29-1.80) and age (p=0.039, risk ratio: 1.019, CI: 1.001-1.037). When replacing sCysC with sCrea, the only significant predictor of mortality was sCrea (p=0.0026; risk ratio 1.20; CI: 1.06-1.36). Increased levels of sCysC can be considered a useful biomarker of cardiovascular mortality in cardiac-surgery patients.

## Introduction

End-stage renal disease requiring dialysis and severe renal disease represent a major risk factor in cardiac-surgery [1].

However, there is another group of patients, showing no obvious clinical and laboratory signs of renal impairment, but who may have already an impaired renal function, such as patients older than 70 years. These patients, representing a significant proportion of those requiring cardiac surgery, have a higher risk of worsening renal function degenerating into a dialysis-dependent renal failure [2]. Postoperative acute renal failure occurs in 5-20% of cases, and about 1% requires dialysis treatment. This serious complication increases the risk of postoperative mortality and morbidity, prolonged hospital stay, higher costs, and requires specific and complex treatments. Therefore, it would be advantageous to identify a marker useful for both renal failure early diagnosis and for cardiovascular (CV) risk stratification. Currently, the most widely method used for renal function monitoring is the evaluation of serum Creatinine (sCrea) levels. However, sCrea levels may be influenced by several factors, such as sex, age, race, metabolism, drugs, protein intake [3,4] and may be inaccurate in several situations, such as in patients with low muscle mass or with fluid overload [5].

In surgical patients, the most sensitive method for diagnosis and staging of AKI (Acute Kidney Injury) would be the comparison between the pre-operative and post-operative sCrea, keeping in mind some previously described limitations [6,7]. Besides the criteria for the RIFLE classification, in association with creatinine levels also the urinary output (UO) can be used to evaluate the loss of kidney function [8].

Cystatin C (CysC) is a non-glycosylated basic protein of the family of cysteine protease inhibitors, is used as a biomarker of renal function and recently has also been evaluated as a potential predictor of the onset or worsening of cardiovascular disease. CysC renal filtration, as well as its plasma concentration, depend essentially on its molecular weight and isoelectric point, and its concentration is influenced by few factors, such as therapy with high doses of glucocorticoids, thyroids dysfunctions, neoplasia or HIV infection [9,10].

The easiness and speed of measurement makes CysC a potentially valuable biomarker for early diagnosis of renal failure, in patients with estimated glomerular filtration within normal limits.

The aim of the study was to assess the potential of preoperative prognostic value of Cystatin C for predicting mortality from cardiovascular causes both in the immediate postoperative period and during a mid-term follow-up.

## RESULTS

The epidemiological and laboratory parameters of the study population are summarized in Table 1. Eight enrolled patients were considered censored because they died for causes other than cardiovascular.

**Table 1:** Epidemiological features of study population.

**Age (years)**	67.70 ± 10.61*
**Sex**	264 males
	160 females
**BMI (kg/m^2^)**	23.2 ± 4.6*
**Hypertension (%)**	85
**Smoke (%)**	19.3
**Mellitus Diabetes (%)**	23.1
**Serum Creatinine (mg/dl)**	1.11±0.86*
**eGFR crea (ml/min/1.73m^2^)**	71.01±21.55*
**Serum Cystatin C (mg/L)**	1.40±0.71*
**eGFRcysC (ml/min)**	58.04±23.54*

*Data is expressed as mean ± Standard Deviation (SD)

We divided the population studied in four subgroups, with respect to both the values of sCysC and sCrea, since there is collinearity between these two filter markers. This subdivision was made to maintain the same proportion among the considered cut-offs (Table 2A).

**Table 2A:** Probability of survival related to laboratory parameters of kidney function and inflammation, type of surgical procedure and age.

**Variables**	**S1**	**S2**	**S3**	**S4**	**p-value effect of category**	**p-value post hoc analysis**
**Cystatin C** **mg/l**	Range <0.81 (n=26)	Range 0.81-1.20 (n=171)	Range 1.21-3.5 (n=214)	Range >3.5 (n=5)	**0.0001**	**S1vs S2 p=0.023** **S1vs S3 p=0.002** **S1vsS4 p=0.0001** **S2vsS3 p=0.001** **S2vsS4 p=0.003** S3vsS4 p=0.140
**Creatinine** **mg/dl**	Range <0.81 (n=116)	Range 0.81-1.20 (n=214)	Range 1.21-3.5 (n=73)	Range >3.5 (n=8)	**0.001**	**S1vs S2 p=0.011** **S1vs S3 p=0.0001** **S1vsS4 p=0.001** **S2vsS3 p=0.005** **S2vsS4 p=0.027** S3vsS4 p=0.516
**Hs-CRP** **mg/l**	Range <3 (n=150)	Range 3.1-6 (n=62)	Range 6.1-9 (n=26)	Range >9 (n=178)	**0.039**	S1vs S2 p= 0.301 S1vs S3 p= 0.577 **S1vsS4 p= 0.004** S2vsS3 p= 0.866 S2vsS4 p= 0.275 S3vsS4 p= 0.373
**Fibrinogen** **mg/l**	Range <351.38 (n=131)	Range 351.38-555.05 (n=186)	Range 555.06-758.73 (n=56)	Range >758.63 (n=12)	0.06	
**Type of performed surgery**	Sur 1	Sur 2	Sur 3	Sur 4	0.263	
**Age** **years**	Range <41 n=10	Range 41-56 n=47	Range 57-72 n=207	Range >72 n=152	0.184	

Sur1= Isolated coronary artery bypass graft procedure

Sur2= Coronary artery bypass graft combined to valve replacement or repair procedure

Sur3= Valve replacement or repair procedure

Sur4= Other surgical procedures (septal defects closure, myxoma resection, atrial fibrillation ablation with Maze procedure)

### Serum cystatin C

**Figure 1:**
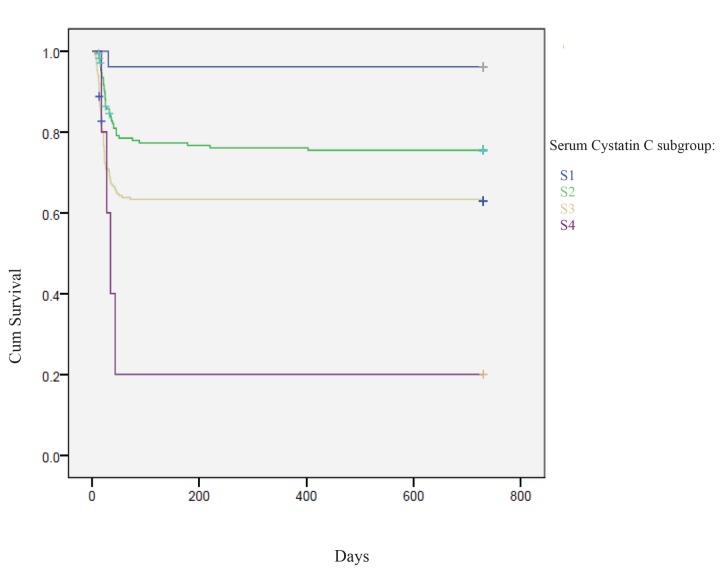
**Kaplan-Meier analysis: mortality percentage in sCysC four subgroups:** S1< 0.81 mg/l, S2= 0.81-1.20 mg/l, S3=1.21-3.5 mg/l and S4 >3.5 mg/l

The survival observed by the Kaplan-Meier analysis in the four subgroups was the following: S1=96.2%, S2=76%, S3= 63.1% and S =20% (Figure 1).

We detected an overall effect of category (p=0.0001) which, in post-hoc testing, appeared to be driven by the following comparisons: S1 vs S2 (p=0.023), S1 vs S3 (p=0.002), S1 vs S4 (p=0.003), S2 vs S3 (p=0.001), S2 vs S4 (p=0.003) (Table 2A).

During the follow-up period, 124 patients (29.4%) died from cardiovascular causes.

### Serum creatinine

**Figure 2:**
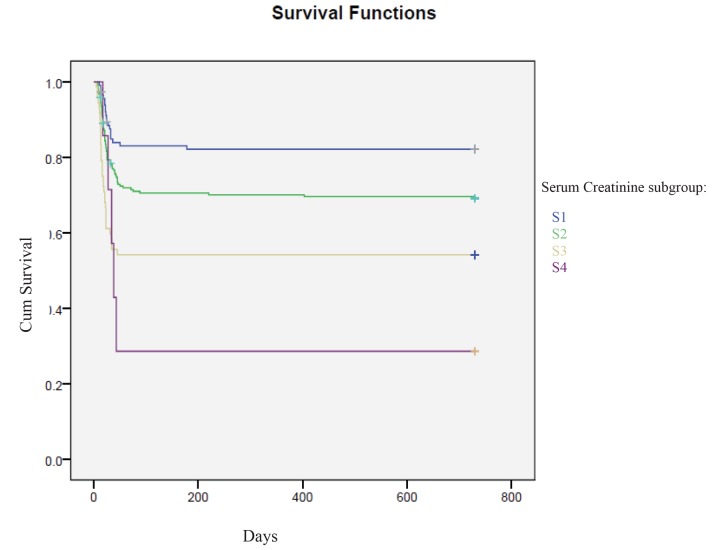
**Kaplan-Meier analysis: mortality percentage in sCrea four subgroups:** S1< 0.81 mg/dl, S2= 0.81-1.20 mg/dl, S3=1.21-3.5 mg/dl and S4 >3.5 mg/dl.

The survival observed by the Kaplan-Meier analysis in the four subgroups was the following: S1=82.6%, S2=69.4%, S3=54.2% and S4=28.6% (Figure 2).

We detected an overall effect of category (p=0.001) which, in post-hoc testing, appeared to be driven by the lowing comparisons: S1 vs S2 (p=0.011), S1 vs S3 (p=0.0001), S1 vs S4 (p=0.001), S2 vs S3. (p = 0.005), S2 vs S4 (p=0.027).

In contrast to what we observed for normal values of sCysC, the Figure 2 shows how even for values ​​of serum creatinine less than 0.81 mg/dl, the probability of survival decreases to 82.6%.

### Inflammatory factors

### *Hs- CRP*

Previous studies showed that inflammation induces the production of cathepsins and increases the plasmatic concentration of CysC (cathepsin inhibitor). Since a direct relationship between the concentration of CysC and CRP (C Reactive Protein) has been previously highlighted, we monitored also the concentration of this inflammatory parameter [11].

So, we examined whether an inflammatory status, monitored by measurement of high sensitivity-CRP (hs-CRP) and fibrinogen, could affect the cardiovascular mortality in these patients (Table 2A).

The survival observed by the Kaplan-Meier analysis in the four subgroups was the following: S1=77.3%, S2=71%, S =73.1% and S4=62.6%.

We detected an overall effect of category (p=0.039) which, in post-hoc testing, appeared to be driven by the comparison S1 vs S4 (p=0.004).

These results seem to show that high levels of hs-CRP, a marker of systemic inflammation, are weakly associated with a significant increase in cardiovascular mortality.

### *Fibrinogen*

The survival observed by the Kaplan-Meier analysis in the four quartiles given above have been the following:

S1 = 78%, S2 = 67%, S3 = 61% and S4 = 67%.

We detected a borderline effect of category (p=0.06) (Table 2A).

### Age

The observed survival (Kaplan-Meier) was: S1 = 80%, S2 = 81%, S3 = 68.6% and S4 = 67.8%.

We detected no significant effect of age category (p=0.184) (Table 2A).

### Sex

The women survival appears 76.3% versus 66.2% for the males group for all the follow-up period.

We detected no significant effect of sex category (p=0.091) (Table 2B).

**Table 2B:** Probability of survival correlated with sex and diabetes.

**Variables**	**Male**	**Female**	**p-value effect of category**
**Sex**	260	156	0.091
	**Yes**	**No**	
**Diabetes**	131	285	0.786

### The presence of type II diabetes mellitus

During the follow-up, the probability of survival remains the same between the two groups of patients (70%). We detected no significant effect of category (p=0.786) (Table 2B).

It would therefore seem that the presence of diabetes mellitus type II is not associated with increased mortality from cardiovascular disease in the study population.

### Type of surgical intervention

The observed survival (Kaplan-Meier) for the different groups were: Int1=66.7%, Int 2=79.5%, Int 3=70.1% and Int4=85.7%.

We detected no significant effect of category (p=0.263) (Table 2A).

### Cox Proportional Hazards Regression

In the first multivariate Cox Proportional Hazard Regression Model (which retained all numerical variables as continuous), the only significant predictors of mortality were Cystatin C (p<0.00001, risk ratio: 1.529, CI: 1.29-1.80) and age (p=0.039, risk ratio: 1.019, CI: 1.001-1.037). When substituting Creatinine for Cystatin C, the only significant predictors of mortality were Creatinine (p=0.0026, risk ratio: 1.204, CI: 1.066-1.359) and age (p=0.040, risk ratio: 1.019, CI: 1.001-1.038). We also compared the two Cox Proportional Hazard Regression Models in terms of the AIC (Akaike's Information Criterium). The model including CysC yielded the lowest AIC, indicating the best fit to the data.

## DISCUSSION

In our study we analyzed cardiac-surgery patients which during the surgical procedure are subjected to extra-corporeal circulation which creates an inflammatory response with production of pro- and anti-inflammatory cytokines [12,^13^].

In addition to the damage created by the circuit, both ischemia and reperfusion contributes significantly to the dysfunction of several organs during the postoperative period. This explains the occurrence of cardiovascular and renal damages. Beside the systemic inflammatory state, the damage due to ischemia and reperfusion alter cardiac function, reducing cardiac output and therefore also perfusion of other organs [14].

Observational studies showed a correlation between systolic blood pressure, hypotension episodes and cardiovascular events [15,16]. In our population, all patients were under medications for hypertension at admission. Moreover, blood pressure values in all the patients were influenced by surgical trauma, postoperative pain and drugs administered during the surgical procedures and postoperative period. Therefore, we did not consider the blood pressure data reliable and accordingly we did not include them among the para-meters examined and the covariates.

Furthermore, these patients have an increased risk of chronic haemodialysis as kidney damage can become irreversible. Unfortunately, when the value of serum creatinine increases, the GFR has already dropped and therefore therapeutic intervention is delayed [7, 17].

Many studies have highlighted the possible role of Cystatin C as an early marker of acute kidney injury and as more reliable as compared to serum creatinine [10, 18].

Other studies have evaluated the usefulness of CysC as a marker of cardiovascular damage or increased risk of onset of cardiovascular disease in the general population [19, 20]. Our study shows that increased levels of sCysC may be considered as predictor of long-term cardiovascular mortality in adult cardiac-surgery patients. In fact, our results obtained evidenced that the cumulative probability of death for cardiovascular causes was significantly higher in patients with enhanced values of sCysC compared to lower levels (observed survival: S4 = 20% vs. S1 = 96.2%). Kaplan-Meier analysis therefore highlighted an overlap between these two markers of kidney function (sCysC and sCrea) (Figures 1 and 2) about the prognostic utility of cardiovascular mortality.

For other parameters exanimated such as age, sex, inflammatory markers (hs-CRP and fibrinogen), presence of type II diabetes mellitus and type of surgical procedure, we detected no significant effect on cardiovascular mortality, except for hs-CRP, whose values seem to be weakly associated with the CV mortality. In the multivariate Cox Proportional Hazard Regression Model, the only significant predictor of mortality was sCysC (p<0,00001; risk ratio 1.529; CI:1.29-1.80).

When substituting sCysC with sCrea, the only significant predictor of mortality was sCrea (p=0.0026; risk ratio 1.20; CI: 1.06-1.36) but with a lower significance as compared to sCysC.

In conclusion, our study shows that sCysC is an independent significant predictor of long-term cardio-vascular events and to provide more consistent and earlier prognostic information than creatinine [21].

The potential usefulness of this molecule, as a clinical marker of cardiovascular risk, require the execution of further studies in a larger population.

## SUBJECTS AND METHODS

We enrolled 424 adults patients (264 men and 160 women, mean age 67.70 ± 10.61 years) in our university hospital’s cardiac-surgery department. We conducted a prospective observational study evaluating mortality for cardiovascular events for two years follow up period.

At admission, all patients were tested for renal function by sCysC (normal range: 0.53-1.20mg/L) and sCrea (normal range: 0.5-1.20 mg/dl). Furthermore, the values of the hs-CRP (normal range: 0.00-0.30mg/dl) and fibrinogen (normal range: 200-400 mg/dl) were also dosed to assess the inflammatory status of patients.

The study protocol complied with the Declaration of Helsinki (1964) and its later amendments or comparable ethical standards. A written fully informed consent was produced by all subjects before the enrolment of the study.

For each patient we evaluated the following additional parameters: Body Mass Index (BMI) using the following formula BMI = weight (kg) /height^2^ (m^2^); presence or absence of diabetes (defined by a history of diabetes, use of oral hypoglycemic drugs or insulin, or a fasting glucose level of 126mg/dl or higher).

The inclusion criteria were:

• age 18-80 years

• patients in the cardiac-surgery department

The exclusion criteria were:

• end stage renal disease in dialysis (haemodialysis or peritoneal dialysis)

• therapy with glucocorticoids, thyroids dysfunctions and systemic inflammatory disease

• active malignancy

• unchecked diabetes mellitus type 2 (Hb glycated > 48 mmoli/moli)

• HIV infection

• BMI < 18.5 Kg/m^2^

The type of surgical intervention performed (Int) were the following: Int 1 = coronary artery bypass graft isolated, Int 2 = coronary artery bypass graft combined to valve replacement or repair procedure, Int 3 = valve replacement or repair procedure, Int 4 = other procedures (correction of septal defects, removal of myxoma, atrial fibrillation ablation with Maze method).

### Laboratory parameters

Blood samples were collected from the antecubital vein in all enrolled patients. The collected serum samples were stored at -20 C° until instrumental analysis.

Serum CysC was measured by a particle-enhanced immuno-nephelometric assay (N Latex Cystatin C, BN ProSpec nephelometer, Siemens Healthcare Diagnostics).

sCrea and hs-CRP measurements were performed by an automated method using Dimension VISTA 1500 (Siemens Healthcare Diagnostics, Milano, Italy).

Fibrinogen was quantified by phototurbimetric method (Ca 7000 Sysmex, Japan).

### GFR calculation

e-GFRcrea was calculated by CKD-EPI (Chronic Kidney Disease Epidemiology Collaboration) equation [22] and eGFRcys was calculated by Cystatin C equation [23].

### Statistical analysis

### *Kaplan-Meier estimates*

In order to evaluate and compare 2-year survival rates in terms of the influence of possible predictors, patients were stratified into categories and Kaplan-Meier estimators were computed and compared for each category. Categorical variables were (Gender, Smoking, Diabetes Mellitus Type II and Type of surgery). In case of continuous predictors (CysC, Creatinine, hs-CRP, Fibrinogen, Age), categories were formed by dividing each candidate predictor into subgroups. The primary outcome was death from cardiovascular disease, and patients who died from causes other than cardiovascular disease were considered censored. When an overall significant effect of category was detected, pairwise comparisons were performed using the Breslow (Generalized Wilcoxon) test. The p-value >0.05 was considered statistically significant.

### *Cox Proportional Hazard Regression*

Additionally, in order to evaluate the simultaneous independent impact of all measured variables on survival we fitted a multivariate Cox-Proportional Hazard Model which employed survival as a dependent variable and continuous variables (sCysC, Age, Fibrinogen, hs-CRP) as well as categorical variables (Gender, Smoking, Diabetes Mellitus Type II, Type of surgery) as predictors. Successively, the same model was fitted substituting sCrea for Cystatin C. This strategy was adopted due to the well know collinearity between these two filtration markers [24] which would have rendered a joint model estimation unstable.
